# Dental management of long-term childhood cancer survivors: a systematic review

**DOI:** 10.1007/s40368-024-00896-5

**Published:** 2024-05-21

**Authors:** K. Seremidi, S. Gizani, G. Dahllöf, M. Barr-Agholme, D. Kloukos, G. Tsilingaridis

**Affiliations:** 1 Department of Pediatric Dentistry, School of Dentistry, National and Kapodistrian University of Athens, Athens, Greece; 2 Division of Orthodontics and Pediatric Dentistry, Department of Dental Medicine, Karolinska Institutet, 14104 Huddinge, Sweden; 3 Center of Pediatric Oral Health, Stockholm, Sweden; 4 Center for Oral Health Services and Research, Mid-Norway (TkMidt), Trondheim, Norway; 5 Department of Orthodontics and Dentofacial Orthopedics, University of Bern, Bern, Switzerland; 6 Department of Orthodontics, 251 Greek Air Force Hospital, Athens, Greece

**Keywords:** Childhood cancer survivors, Antineoplastic treatment, Dental late effects, Dental management

## Abstract

**Purpose:**

Critically review and summarise existing knowledge on prevalence of oral, dental, and craniofacial side-effects of antineoplastic treatment in childhood cancer survivors (CCS).

**Methods:**

A literature search was conducted for studies reporting on children aged 4–19 years treated for any type of malignancy up to the age of 15 years and for whom, at the time of the examination, more than 8 months have elapsed since the end of treatment. Data regarding dental late effects on teeth and craniofacial complex were collected and mean prevalence of each defect was reported.

**Results:**

From the 800 articles identified, 17 studies fulfilled inclusion criteria and were included. A total of 983 CCS were examined, with the total number of healthy controls being 1266 children. Haematological malignancy was the most prevalent diagnosis with the age at diagnosis ranging between 0–15 years. Multiple antineoplastic protocols were implemented with the elapsed time being 8 months up to 17 years. One-third of CCS experienced at least one late effect, with corresponding value for the control group being below 25%. Among the defects identified clinically, microdontia, hypodontia and enamel developmental defects were recorded in 1/4 of CCS. Impaired root growth and agenesis were the two defects mostly recorded radiographically. The effect on dental maturity and on salivary glands was unclear.

**Conclusion:**

CCS are at risk of developing dental late effects because of their disease and its treatment and therefore, routine periodic examinations are essential to record their development and provide comprehensive oral healthcare.

## Introduction

The overall 5-year survival rate from childhood cancer has improved and now exceeds 80% in developed countries (Winther et al. 2015). With a simultaneous decrease in late mortality the number of long-term survivors is steadily increasing (Fidler et al. 2016). Because of their curative treatment-related exposures, survivors of childhood cancer are at increased risk for a broad range of chronic health conditions (CHC). A recent survey by Bhakta et al. (2017) showed that the cumulative incidence of CHCs at age 50 years was 99·9% and 96·0% for more severe conditions. By age 50 years, a survivor had experienced, on average, 17·1 CHCs of any grade, of which 4·7 were more severe. Second neoplasms, spinal disorders, and cardiopulmonary disease were major contributors to the excess total cumulative burden. There is a constant development of treatment protocols for childhood cancers, where treatment intensity for cancers with a relatively good prognosis has decreased to prevent morbidity, whereas conversely treatment has intensified for cancers with poor prognoses to improve survival (Fidler et al. 2016). Recent follow-up studies of long-term survivors of childhood cancer show that more recently treated patients not only have a significantly lower rate of late mortality due to progression or recurrence of their primary tumour but a reduced rate of mortality due to treatment-related late effects such as second malignancies and cardiopulmonary conditions (Armstrong et al. 2016). None of these follow-up studies include oral, dental, and craniofacial adverse effect of therapy.

Individuals treated for childhood cancer experience a wide range of severe complications also in the oral cavity, regarding dental and craniofacial development. Childhood cancer survivors (CCS) have a higher prevalence of oral and dental abnormalities than controls, type of cancer treatment, socioeconomic factors, and access to oral health care contribute to the prevalence of dental abnormalities (Patni et al. 2023). In a systematic review, Gawade et al. (2014) reported that CCS had a higher prevalence of dental caries, as well as strong evidence to support an association between chemotherapy and dental developmental abnormalities, such as dental agenesis, dental hypoplasia, root stunting, and enamel hypoplasia. The combination of chemotherapy with radiation therapy or conditioning with total body irradiation in stem cell transplant recipients confer an even higher risk of oral, dental, and craniofacial disturbances.

Three systematic reviews have been published regarding long-term dental and oral complication in survivors of childhood cancer (Gawade et al. 2014; Busenhart et al. 2018; Seremidi et al. 2019). The study by Gawade et al. (2014) included studies published up to 2012, but did not include a meta-analysis, Busenhart et al. (2018) included only children treated with chemotherapy protocols in studies published up to 2016 and Seremedi et al. (2019) included 16 studies published up to 2018.

The rationale for this systematic review and potential meta-analysis is that recent long-term follow-up of late effects in survivors of childhood cancer aiming to characterise the overall health burden have not included oral, dental, or craniofacial side-effects (Bhakta et al. 2017; Erdman et al. 2021; Chung et al. 2022). Furthermore, that the systematic reviews on oral, dental, and craniofacial side-effects published have included studies published 2018 or earlier, we have identified several studies published since then. So, the aim of the present review was to summarise and critically appraise existing knowledge regarding prevalence of oral, dental, and craniofacial side-effects of antineoplastic treatment. Secondary objective was to summarise evidence on dental and oral care in long-term CCS, in terms of both self/home-care measures and dental rehabilitation in the practice setting.

## Materials and methods

The protocol was submitted to the PROSPERO international prospective register of systematic reviews hosted by the National Institute for Health Research (NIHR), University of York, UK, Center for Reviews and Dissemination. The CRD42023399543 identification number was allocated.

### Reporting format

The Preferred Reporting Items for Systematic Reviews and Meta-Analyses (PRISMA) were adopted, and the review was planned, conducted and reported according to the standards of quality for reporting systematic reviews (Page et al. 2021). PICO methodology (Table 1) was utilised to formulate the research question: “What are the long-term effects of antineoplastic treatment on the craniofacial complex and what are the challenges in the dental management of long-term childhood cancer survivors?”.

**Table 1:** PICO criteria

Criteria	Definition
Population	Children and adolescents up to the age of 19 years at the day of examination that have undergone antineoplastic treatment up to the age of 15 years and are in remission for at least 8 months
Intervention	Any type of antineoplastic treatment administered solely or in combination (chemotherapy, radiotherapy, Haemopoietic stem cell transplantation)
Comparators	Presence or absence of a treated or untreated control group
Outcomes	1. Primary outcomes A. Oral health B. Dental caries (DMFT/dmft, prevalence or incidence of decayed teeth) C. Oral hygiene (Gingival index, plaque index, OHI, CPI) D. Prevalence of crown defects (microdontia, macrodontia, hypodontia, hypoplasia, malformed teeth, discoloured teeth) E. Prevalence of root defects (impaired root growth, arrested root growth, V-shaped roots, taurodontism, premature apical closure and tooth agenesis) F. Effect on dental maturity G. Effect on salivary glands (salivary flow rate, buffer capacity, microbial counts) H. Preventive strategies administered by the clinician for home and practice use I. Dental care (restorative, orthodontic, oral surgery, prosthodontic rehabilitation) 2. Secondary outcomes Effect of treatment on patient’s long-term health-related quality of life and oral health-related quality of life Use of dental services and compliance with follow-ups Knowledge and attitudes of medical doctors and dentists regarding late effects and dental management of CCS

### Inclusion and exclusion criteria

Eligible were case–control, cross-sectional, observational and cohort studies with a retrospective design on children and adolescents:

- aged 4–19 years old at the time of dental examination
- with a history of malignancy, treated with various protocols (chemotherapy, radiotherapy, haemopoietic stem cell transplantation) from birth up to the age of 15 years
- for whom, at the time of the examination, more than 8 months have elapsed since the end of antineoplastic treatment.

Case reports and case series were also considered eligible for summarising evidence on dental therapeutic management of CCS.

Studies reporting on CCS aged > 19 years of age, that have been treated after the age of 15 years, and with active disease or under treatment were excluded. Excluded were also studies reporting on effects of antineoplastic treatment detected during treatment, immediately after or < 8 months after treatment cessation and on the effect on any other organ apart from teeth and the craniofacial complex. Finally, studies written in a non-English language were excluded.

### Search strategy

A literature search was conducted in the following electronic databases: Medline/Pubmed, Embase, LILACS and The Cochrane Library [Cochrane Database of Systematic Reviews, Cochrane Central Register of Controlled Trials (CENTRAL), Cochrane Methodology Register] (Appendix 1).

Unpublished literature on ClinicalTrials.gov (www.clinicaltrials.gov), the National Research Register (www.controlled-trials.com) and grey literature and bibliographies of published articles was also searched to identify studies not identified previously. The reference lists of all eligible studies and other previously published systematic reviews on the topic were screened manually for other potentially eligible studies.

In all searches no publication date restrictions were applied.

### Study selection

The titles and/or abstracts of all studies retrieved from the search, and those from additional sources, were screened independently by two review authors. After exclusion of the non-eligible full-texts of all studies considered as eligible by any of the authors were assessed independently and in duplicate. Any discrepancies and disagreements were resolved thorough discussion by the two reviewers. Should this not be possible, a third author was consulted.

### Data extraction

Data were extracted independently and in duplicate by two reviewers in specifically designed forms. For each study the following information were recorded: publication details (authors, year of publication, design), sample characteristics (sample size, control group, participants age at examination, diagnosis, age at diagnosis, treatment undertaken, post-treatment follow-up time), outcome evaluated including methods of assessment. For studies with missing/unclear data, the authors were contacted via e-mail for further clarifications and in cases of no response within a period of 15 days, the study was excluded.

In studies reporting on dental management age at presentation, diagnosis and age at diagnosis, dental late effects documented and dental treatment undertaken were recorded for each case.

### Quality assessment

Risk of bias was assessed by two reviewers independently, using the Newcastle–Ottawa scale (NOS) adopted for case–control, cross-sectional and cohort studies (Wells et al. 2010). For each study, presence of bias was assessed in three different domains named: (a) sample selection, (b) comparability and (c) outcome. Each domain gets a score with studies scoring > 5 stars for cross-sectional and cohort studies and > 7 stars for case–control studies considered as being “of good quality”. Quality of case reports included was assessed using the JBI critical appraisal checklist (Gagnier et al. 2013).

### Data analysis

Collected data for each outcome that was a numerical index were presented as mean, minimum and maximum values calculated from values reported from all included studies. For clinical and radiographic developmental dental defects, mean prevalence was calculated from the prevalence in studies reporting on them. Meta-analyses were planned to be conducted with studies reporting similar interventions and comparable outcomes, i.e. in the case of limited methodological and clinical heterogeneity. Data were planned to be analysed with Review Manager 5.4 [Review Manager (RevMan), Version 5.4, The Cochrane Collaboration, Copenhagen, 2020].

### Heterogeneity

Clinical and methodological heterogeneity were assessed by examining the characteristics of the studies, the similarity between the types of participants, the interventions, and the outcomes as specified in the inclusion criteria for considering studies for this review.

Statistical heterogeneity will be assessed using a Chi^2^ test and the *I*^2^ statistic, where *I*^2^ values between 50–90% indicate substantial heterogeneity.

### Assessment of reporting bias

In the presence of more than 10 studies in a meta-analysis, the possible presence of publication bias would be investigated for the primary outcomes.

### Subgroup analysis

In the case of sufficient data, subgroup analyses would be conducted to explore the influence of study or patient characteristics such as gender and/or age, type of malignancy, age at diagnosis, treatment protocol administered and time that has elapsed since end of treatment.

### Sensitivity analysis

Analysis of studies stratified by design or by risk of bias (i.e. overall low risk versus high risk) were planned to be explored for similar or different results.

### Unit of analysis

Some of the included studies presented data from repeated observations on participants, which could lead to unit-of-analysis errors and for that reason the advice in Sect. 9.3.4 of the Cochrane Handbook for Systematic Reviews of Interventions was followed (Higgins and Green 2011).

## Results

### Search results

**Fig. 1:**
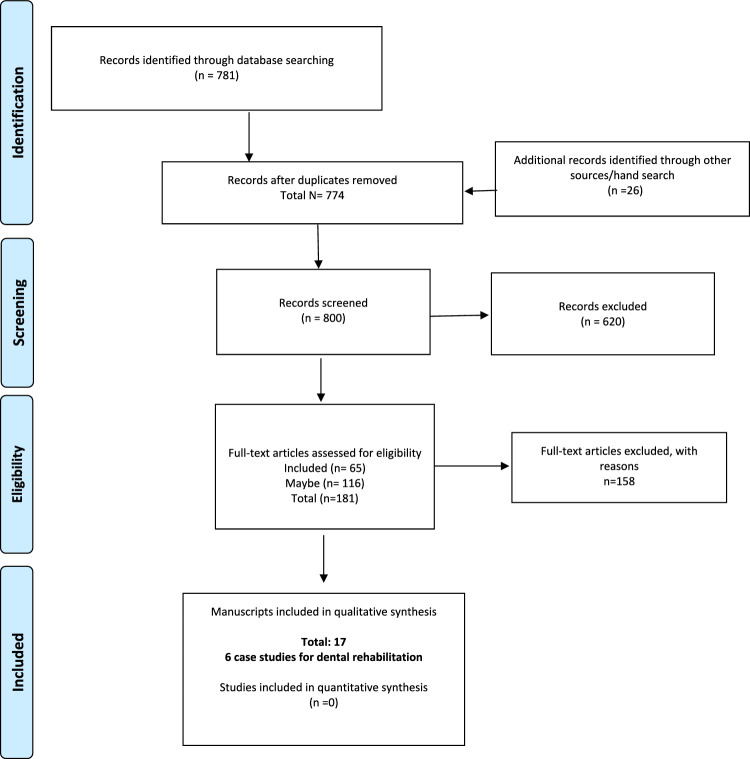
PRISMA flow diagram

As presented in the PRISMA flowchart (Fig. 1), 781 articles were initially identified to which another 26 were added following hand search. After duplicates removal, a total of 800 articles underwent title and abstract screening, of which 620 were excluded and 181 articles were retrieved for full-text appraisal. From those 158 were excluded with reasons (Table 2), leaving a total of 17 retrospective studies and 6 case studies finally included.

**Table 2:** List of excluded studies and the rationale behind

Reasons for exclusion (for studies with more than 1 reason, only 1 reason will be given)	Title, year and authors	Number of papers
Wrong population	Atif et al., 2022; Guagnano et al., 2022; Halperson et al., 2022; Hamilton et al., 2022; Stolze et al., 2022; Seremidi et al., 2021; Stolze et al., 2021; Almendra Mattos et al., 2020; Frascino et al., 2020; Quispe et al., 2019; Loves et al., 2019; Mattos et al., 2019; Proc et al., 2019; Kang et al., 2018; Wani et al., 2018; Balcerek et al., 2017; Tanaka et al., 2017; Olszewska et al., 2016; Willberg et al., 2016; Thomaz et al., 2013; Hegde et al., 2011; Perwein et al., 2011; Hutton et al., 2010; van Der Pas-van Voskuilen et al., 2009; Cubukçu and Sevinir, 2008; Martin et al., 2008; Wogelius et al., 2008; Avşar et al., 2007; Oeffinger et al., 2006; Hölttä et al., 2005a; Hölttä et al., 2005b; Yeazel et al., 2004; Minicucci et al., 2003; Kahnberg et al., 2002; Pajari et al., 2001; Yalman et al., 2001; Alpaslan et al., 1999; Schwarz et al., 1999; Duggal et al., 1997; Uderzo et al., 1997; Maguire et al., 1996; Dens et al., 1995; Cacchillo et al., 1993; Nunn et al., 1991; Larson et al., 1990; Sonis et al., 1990; Dahllöf et al., 1989; Pajari et al., 1988; Maguire et al., 1987; Jaffe et al., 1984; Welbury et al., 1984; Brown et al., 1975	5 2
Wrong outcome	Jodlowska et al., 2022; Kim et al., 2022; Larsen et al., 2022; Latoch et al., 2022; Immonen et al., 2021; Lo et al., 2021; George et al., 2020; Elzembely et al., 2019; Valentini et al., 2018; Hartnett et al., 2017; Sahai et al., 2017; Garfein et al., 2015; Mitus-Kenig et al., 2015; Neil et al., 2015; Cohen et al., 2014; Qureshi et al., 2014; Saha et al., 2014; Smith et al., 2014; Casillas et al., 2011; Hsieh et al., 2011; Pedersen et al., 2012; Wogelius et al., 2009; Cubukçu and Sevinir, 2008; Hobbie et al., 2008; Lal et al., 2007; Flandin et al., 2006; Kupeli et al., 2006; Chen et al., 2004; da Fonseca 2004; Duggal et al., 2003; Dahllöf et al., 2001; Paulino et al., 2000; Raney et al., 1999; Clarkson et al., 1998; Niethammer et al., 1998; Kinirons et al., 1995; Mulhern et al., 1995; Fayle et al., 1992; Purdell-Lewis et al., 1988; MacLeod et al., 1987; Rosenberg et al., 1987; Fromm et al., 1986	42
Wrong study design (i.e. case report)	Rahul et al., 2023; Bledsaw et al., 2022; Peyam et al., 2022; Rahul et al., 2021; Hoogeveen et al., 2020; Ritwik et al., 2020; Gunen et al., 2018; Weyl-Ben-Arush et al., 2017; Owosho et al., 2016; Javed et al., 2012; Venkataraghavan et al., 2013; Najafi et al., 2011; Zarina et al., 2005; Cheng et al., 2000; Pajari et al., 1996; Goho 1993; Sanders 1991; Williams et al., 1991; Berkowitz et al., 1989; Sanders et al., 1989	20
Wrong publication type (i.e. study protocol, conference proceeding)	Sidi Omar et al., 2021; Brignardello-Petersen et al., 2019; Horner et al., 2019; Psoter et al., 2019; Morais et al., 2014; Thouvenin-Doulet et al., 2015; Carillo et al., 2014; Effinger et al., 2014; Epstein et al., 2012; Wogelius et al., 2011; Xavier et al., 2010; Dahllöf et al., 2008; Elting et al., 2008; Dickerman 2007; Ayers et al., 2000; Singh et al., 1996; Kaste et al., 1994; Fleming et al., 1991; Sanders et al., 1990; Leventhal et al., 1989	20
Non-English papers	Markov et al., 2020; Mladosievicova et al., 2015; Akharzouz et al., 2013; van Der Pas-van Voskuilen et al., 2010; Balwierz et al., 2006; Alberth et al., 2002; Peretz et al., 2001; Maire et al., 1999; Nikoui et al., 1996; Holtgrave et al., 1995; Nawrocki et al., 1995; Majorana et al., 1994; Bocca et al., 1990; Ikeda et al., 1990	14
Not adequate information	Proc et al., 2022; Cetiner et al., 2019; Lauritano et al., 2012; Vasconcelos et al., 2009; Kaste et al., 1998; Näsman et al., 1997	6
Not found	Makdissi et al., 2004; Doğan et al., 2001; Jones et al., 1992; Donohue et al., 1965	4
Total		158

For studies reporting data not in the form described in the inclusion criteria (e.g. age at diagnosis or age at dental examination as mean and not range), attempts were made to contact authors to request for raw data to decide upon inclusion.

### Study characteristics

The characteristics of the included studies are summarised in Table 3. From the studies included into the analysis, six were cross-sectional (Shum et al. 2020; Proc et al. 2016; Flores et al. 2015; Cubukçu and Sevinir 2008; Lopes et al. 2006; Marec-Berard et al. 2005), ten case–control studies (Mitsea et al. 2022; Proc et al. 2021; Kılınç et al. 2019; Olczak-Kowalczyk et al. 2018; Bagattoni et al. 2014; Nemeth et al. 2013, 2014; Cubukcu et al. 2012; Oğuz et al. 2004; Näsman et al. 1994) and one cohort study (Shayani et al. 2022). They were all published in English from 1994 until 2022. The size of the studies’ sample ranged from 25 to 137 children, yielding a total of 983 patients that were long-term survivors. A control sample was included in all but four studies, ranging from 26 to 521 patients, yielding a total of 1266 healthy children. The control sample consisted of age and gender matched healthy subjects, or healthy siblings (Kılınç et al. 2019).

**Table 3:** Study characteristics of all included studies (*n* = 17)

Author/year	Study design	Sample size (*N*)	Control sample (*N*)	Age at oral exam	Diagnosis	Age at diagnosis	Treatment	HSCT	Post-treatment follow-up time	Outcome evaluated	
										Clinical	Radiographic
Mitsea et al. (2022)	Case–control	72	72	5–16 years	Haematological, CNS tumour, ST	11 months–10 years	CHX (*n* = 51) CHX + RDT (*n* = 22)	No	> 1 year		Dental maturity
Shayani et al. (2022)	Cohort	54	46	4–13 years	ALL	3–13 years	CHX	No	2 years	Dental caries	
Proc et al. (2021)	Case–control	59	177	4–16 years	Leukaemia, lymphoma, brain tumour, WT, hepatoblastoma, neuroblastoma, solid tumours	1mnth – 10.3 years	CHX (*n* = 34) RDT (*n* = 22)	8 patients	8 months–10 years	Crown defects	Dental maturity, root defects
Shum et al. (2020)	Cross-sectional	68		14–16 years	Leukaemia, lymphoma, retinoblastoma, kidney, CNS tumour, RMS, other	< 10 years	CHX RDT (*n* = 25)	12 patients	14.9 mean		Agenesis
Kilinc et al. (2019)	Case–control	93	73 siblings	8–13 years	Leukaemia, lymphoma, Langerhans cell, NBL, RTB, renal tumour, hepatic tumour, CNS tumour, germ cell tumour, soft-tissue sarcoma	9 months–7 years	CHX (*n* = 58) CHX + RDT (*n* = 35)	No	5–8 years	Crown defects	Root defects
Olczak-Kowalczyk et al. (2018)	Case–control	60	60	6–18 years	Medulloblastoma, WT, RMS, Burkitt’s lymphoma, NBL, Ewing’s sarcoma, other	9 months–15 years	CHX	No	> 1 year	Dental caries	
Proc et al. (2016)	Cross-sectional	51	521	5–18 years	ALL, WT, NBL, RMS, brain tumour, lymphoma, germinal tumour, other	1 month–15 years	CHX (*n* = 50) RDT (*n* = 8)	11 patients	8 months–12 years	Crown defects	Root defects, agenesis, hypodontia
Flores et al. (2015)	Cross-sectional	50	–	6–15 years	NR	1 month–13 years	CHX CHX + RDT	No	> 1 year		Dental maturity
Bagattoni et al. (2014)	Case–control	25	26	7–19 years	ALL, lymphohistiocytosis, medulloblastoma, NBL, sarcomas	0–7 years	CHX CHX + RDT	2 patients	3–13 years	Dental caries, enamel developmental defects, crown defects	Root defects, agenesis, dental maturity
Nemeth et al. (2014)	Case–control	38	40	12 years	Lymphoma, NBL, soft-tissue sarcoma, osteosarcoma, HL	2.5 – 6 years	CHX	No	Mean 6.9 years	Salivary flow rate, Buffer capacity	
Nemeth et al. (2013)	Case–control	38	40	12 years	NR	31 months–6 years	CHX	No	Mean 6.9 years	Oral health, dental caries,	Root defects, agenesis
Cubukcu et al. (2012)	Case–control	37	37	6–15 years	ST, lymphoma	0–7 years	CHX (*n* = 27) CHX + RDT (*n* = 10)	No	> 5 years	Crown defects	Agenesis, root defects
Cubukçu and Sevinir (2008)	Case–control	62	62	6–19 years	Lymphomas, WT, RTB, RMS, carcinoma, histiocytoma, other	5 months –14 years	CHX (*n* = 62) CHX + RDT (*n* = 16)	No	Mean time 5 years	Caries	
Lopes et al. (2006)	Cross-sectional	137	–	6–12 years	Leukaemia, lymphomas, ST	0–10 years	CHX (*n* = 92) CHX + RDT (*n* = 45)	No	> 1 year	Crown defects	Root defects
Marec-Berard et al. (2005)	Cross-sectional	27		4–13 years	Nephroblastoma	8 months–8.6 years	CHX	No	> 24 months	Crown defects	Root defects, agenesis
Oğuz et al. (2004)	Case–control	36	36	4–17.6 years	NHL	3.2–15 years	CHX	No	1–6.2 years	Oral health, dental caries, crown defects	Root defects, agenesis
Näsman et al. (1994)	Case–control	76	76	> 12 years	Leukaemia, lymphomas, NBL, CNS tumours, WT, sarcomas, other tumours	1–8 years	CHX (*n* = 57) CHX + TBI (*n* = 19)	19 patients	3–8 years for HSCT 3–17 years for CHX	Dental caries, saliva, enamel developmental defects	Root defects

*HSCT* haemopoietic stem cell transplantation, *CNS* central nervous system, *ST* solid tumours, *All* acute lymphoblastic leukaemia, *WT* Will’s tumour, *RMS* rhabdomyosarcoma, *NBL* neuroblastoma, *HL* Hodgkin’s lymphoma, *NHL* non-Hodgkin’s lymphoma, *CHX* chemotherapy, *RDT* radiotherapy, *TBI* total body irradiation, *NR* not reported

The age of the participants at the day of dental examination ranged between 4–19 years, while the age at cancer diagnosis ranged between 0 and 15 years. Diagnosis in most cases included more than one type of malignancy with haematological malignancies being the most common, followed by solid tumours. Multiple antineoplastic protocols were implemented with chemotherapy being the type of treatment used in all studies. Concomitant radiotherapy was applied in 10 studies (Mitsea et al. 2022; Proc et al. 2021; Shum et al. 2020; Kılınç et al. 2019; Proc et al. 2016; Flores et al. 2015; Bagattoni et al. 2014; Cubukçu and Sevinir 2008; Cubukcu et al. 2012; Lopes et al. 2006) with TBI in one (Näsman et al. 1994. In five studies (Proc et al. 2021; Shum et al. 2020; Proc et al. 2016; Bagattoni et al. 2014; Näsman et al. 1994) part of the sample had undergone HSCT, with the number of patients ranging from 2 to 19. Finally, the time that has elapsed from the end of treatment ranged from 8 months to 17 years.

Regarding the outcomes evaluated, eight studies (Shayani et al. 2022; Olczak-Kowalczyk et al. 2018; Bagattoni et al. 2014; Nemeth et al. 2013, 2014; Cubukcu et al. 2012; Oğuz et al. 2004; Näsman et al. 1994) reported on dental caries, one on oral hygiene, one on gingival and plaque index and one on periodontal status. Eleven studies (Proc et al. 2021; Shum et al. 2020; Kılınç et al. 2019; Proc et al. 2016; Bagattoni et al. 2014; Nemeth et al. 2013; Cubukcu et al. 2012; Lopes et al. 2006; Marec-Berard et al. 2005; Oğuz et al. 2004; Näsman et al. 1994) reported on clinical findings including crown defects, hypodontia and enamel developmental defects, and on radiographic findings as the prevalence of root defects, premature apical closure, agenesis and delayed eruption. Finally, four studies (Mitsea et al. 2022; Proc et al. 2021; Flores et al. 2015; Bagattoni et al. 2014) reported on dental maturity and two studies (Nemeth et al. 2014; Näsman et al. 1994) on salivary gland functions through flow rate, buffer capacity and microbial counts.

### Quality assessment

Tables 4 and 5 present the summary findings of the quality assessment for potential risk of bias in all included studies. Overall, most studies regardless of their design were considered as being of low risk of bias. Specifically, all but one case–control study (Mitsea et al. 2022), scored excellent in the selection domain, indicating that study and control samples are representative of the population under study. In the study by Mitsea et al. (2022), selection domain got three out of four stars due to potential bias regarding representativeness of the case. Three studies (Mitsea et el. 2022; Proc et al. 2021; Olczak-Kowalczyk et al. 2018) lost one star in the comparability domain as reviewers judged that comparability of cases and controls was based on the most important factor and not on any additional factors that could have an impact on the outcome. Exposure domain was excellent for all cases. The cohort study (Shayani et al. 2022), dropped a star in the selection domain as it failed to demonstrate that the outcome was not present at the beginning of the study and one in the outcome domain due to the lack of blind independent assessment. Finally, for the cross-sectional studies half (Proc et al. 2016; Lopes et al. 2006; Marec-Berard et al. 2005) were considered of low risk of bias. The remaining were down scored for potential risk of selection bias regarding the non-respondent rate and the lack of independent blind assessment of the outcome.

**Table 4:** Quality assessment for case–control studies, using the Newcastle–Ottawa Scale tool

Study	***Selection***	***Comparability***	***Exposure***	***Total***
Mitsea et al. (2022)	***	*	***	7/9
Proc et al. (2021)	****	*	***	8/9
Kilinc et al. (2019)	****	**	***	9/9
Olczak-Kowalczyk et al. (2018)	****	*	***	8/9
Bagattoni et al. (2014)	****	**	***	9/9
Nemeth et al. (2014)	****	**	***	9/9
Nemeth et al. (2013)	****	**	***	9/9
Cubukcu et al. (2012)	****	**	***	9/9
Oğuz et al. (2004)	****	**	***	9/9
Näsman et al. (1994)	****	**	***	9/9

**Table 5:** Quality assessment for cross-sectional and cohort studies, using the Newcastle–Ottawa Scale tool

Study	Selection	Comparability	Outcome	Total
Cross-sectional studies				
Shum et al. (2020)	**	*		3/7
Proc et al. (2016)	***	**	*	6/7
Flores et al. (2015)	**	*	*	4/7
Cubukçu and Sevinir (2008)	*			1/7
Lopes et al. (2006)	***	**	**	7/7
Marec-Berard et al. (2005)	***	**		5/7
Cohort studies				
Shayani et al. (2022)	***	**	**	7/9

All case reports were of relatively high quality, as they clearly described most of the characteristics related to the presentation, diagnosis, treatment, and follow-up of the cases. Despite half lacking detailed presentation of patients’ main characteristics related to diagnosis and treatment protocols followed, dental defects detected, and patients’ rehabilitation were clearly mentioned, highlighting the uniqueness of each case. They all summarised key points and provide good guidance for clinicians when they deal with these patients (Table 6).

**Table 6:** Quality assessment of case reports included, based on the description of specific characteristics

	Demographic characteristics clearly described	History clearly described and presented as a timeline	Clinical condition on presentation clearly described	Diagnostic tests or assessment methods and the results clearly described	Intervention(s) or treatment procedure(s) clearly described	Post-intervention clinical condition clearly described	Adverse events (harms) or unanticipated events identified and described	Provide takeaway lessons
Chang and Lin (2021)	+/−	+/−	+	+	+	+	++	+
Liu et al. (2021)	+/−	+/−	+	+	+	+/−	+	+
King (2019)	+	+/−	+	+	+	+/−	+/−	+
Michalak et al. (2019)	+/−	+/−	+	+	+	+	+	+
Kotsiomiti et al. (2013)	+	+	+	+	+	+	+	+
Zwetchkenbaum and Oh (2007)	+	+	+	+	+	+	+	+

+  yes, − no, +/− unclear, *N/A* not applicable

### Qualitative synthesis

Overall, one-third of CCS experienced at least one late effect, with corresponding value for the control group being below 25% in most cases. Root abnormalities and agenesis were the two most common defects recorded among all patients examined.

#### Oral health

**Fig. 2:**
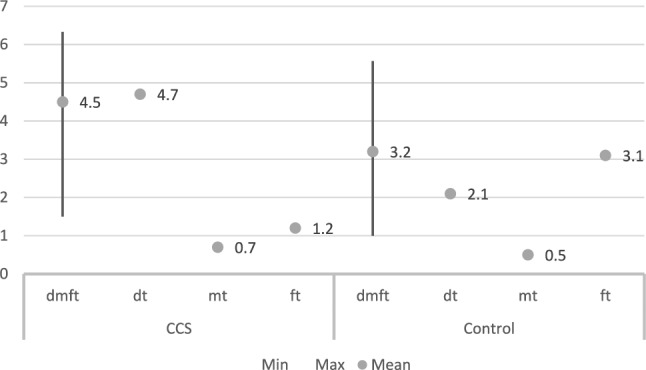
Overall values (minimum, maximum, mean) for caries index in primary dentition as calculated from the included studies for survivors and healthy controls

**Fig. 3:**
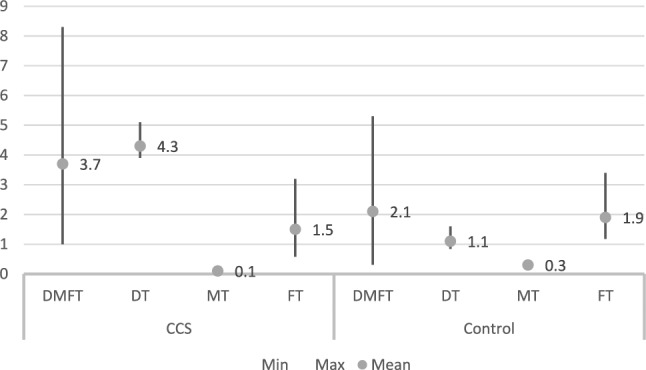
Overall values (minimum, maximum, mean) for caries index in permanent dentition calculated from the included studies for survivors and healthy controls

The effect of the disease and its treatment on oral health, recorded as oral hygiene or dental caries was recorded in eight studies (Table 7) all of which included a control group. Overall mean dmft value for CCS was 4.5, ranging between 1.5 and 6.33 (Fig. 2). Corresponding values for permanent dentition were 3.7, with 1 being the minimum value and 8.3 the maximum value recorded (Fig. 3). Overall mean values for the control group were 3.2 and 2.1, respectively. Considering the different components of the index, for CCS decayed teeth had the highest mean value of all for both primary (4.7) and permanent dentition (4.3), while filled teeth was the component with the highest mean value in the control group (ft = 3.1, FT = 1.9). Values for DMFS/dmfs reported for CCS were higher than those recorded for the control group.

**Table 7:** Mean values recorded in all included studies for dental caries, oral hygiene, gingival and periodontal indices

Author/year	Dental caries		Oral hygiene		GI		PI		CPI	
	CCS	Control	CCS	Control	CCS	Control	CCS	Control	CCS	Control
Shayani et al. (2022)	dmft: 4.26 DMFT: 1.58	dmft: 3 DMFT: 0.31								
Olczak-Kowalczyk et al. (2018)	dmft: 6.33 dt: 4.67 mt: 0.7 ft: 1.2 DMFT: 8.3 DT: 5.1 MT: 0.15 FT: 3.2	dmft: 5.57 dt: 2.07 mt: 0.47 ft: 3.1 DMFT: 5.3 DT: 1.6 MT: 0.4 FT: 3.4								
Bagattoni et al. (2014)	dmft: 1.5 DMFT: 1	dmft/: 1 DMFT: 1.2								
Nemeth et al. (2013)	DMFT: 4.61 DT: 3.97 MT: 0.05 FT: 0.58	DMFT: 2.21 DT: 0.84 MT: 0.18 FT: 1.18	1.53	0.99					Healthy: 53% Bleeding: 42% calculus: 5.3%	Healthy: 53% Bleeding: 40% calculus: 7.5%
Cubukçu and Sevinir (2008)	dmft: 5.8 DMFT: 2.1	dmft: 3.4 DMFT: 1.6								
Oğuz et al. (2004)	dmft/DMFT: 6.25 dmfs/DMFS: 9.52	dmft/DMFT: 4.8 dmfs/DMFS: 7.05			1.09	0.85	1.49	0.90		
Näsman et al. (1994)	CHX: DFS: 5.1 DS:1.3 HSCT: DFS: 3.5 DS: 0.6	DFS: 2.7 DS: 0.3								

*DMFT* decayed, missing, filled teeth index, *DMFS* decayed, missing, filled surfaces index, *DT* decayed teeth, *MT* missing teeth, *FT* filled teeth, *GI* gingival index, *PI* plaque index, *CPI* community periodontal index, *CCS* childhood cancer survivors, *CHX* chemotherapy, *HSCT* haemopoietic stem cell transplantation

In the two studies (Nemeth et al. 2013; Oğuz et al. 2004) that report on oral hygiene and periodontal health CCS had higher mean values for oral hygiene (1.53), gingival (1.09) and plaque index (1.49) as compared to heathy controls (0.99, 0.85 and 0.90, respectively). Values for CPI were comparable between the groups, with survivors showing slightly higher prevalence of gingival bleeding (42% Vs 40%) and slightly lower for calculus (5.3% vs 7.5%).

#### Late defects recorded clinically

**Fig. 4:**
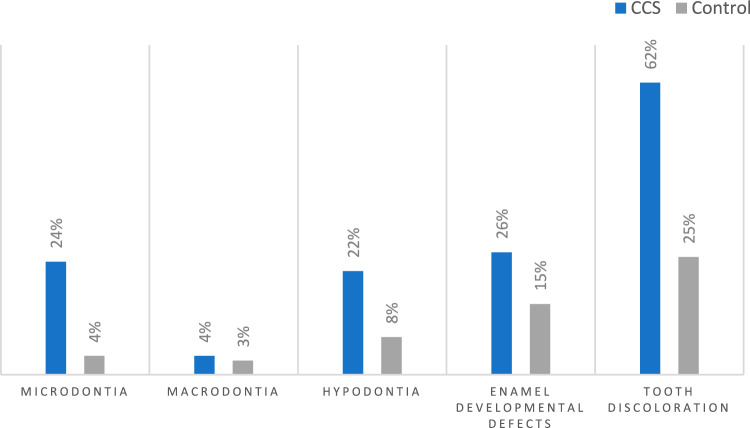
Overall prevalence of dental late defects recorded clinically in all included studies

Clinical dental late effects were recorded in 11 studies and their prevalence in each study is presented in Table 8. In the study group discolouration was the most prevalent defect (62%), while microdontia, hypodontia and enamel developmental defects were recorded in ¼ of the patients (Fig. 4). Overall prevalence for the control group was lower ranging from 3% for macrodontia to 25% for tooth discolouration.

**Table 8:** Frequency (number of patients) and prevalence of clinical dental late effects as reported in included studies

Authors/year	Microdontia		Macrodontia		Hypodontia		Enamel developmental defects		Discolouration	
	No. of patients (%)		No. of patients (%)		No. of patients (%)		No. of patients (%)		No. of patients (%)	
	CCS	Control	CCS	Control	CCS	Control	CCS	Control	CCS	Control
Proc et al. (2021)	18 (30)				16 (27)					
Shum et al. (2020)	4 (6)									
Kilinc et al. (2019)	60 (65)	–			21 (23)	–	22 (23)	7 (10)		
Proc et al. (2016)	22 (36)	15 (3)			14 (23)	44 (8)				
Bagattoni et al. (2014)	6 (24)	0 (0)					5 (20)	1 (4)	14 (56)	–
Nemeth et al. (2013)	12 (32)	0 (0)	1 (3)	1 (3)						
Cubukcu et al. (2012)	5 (14)	5 (14)								
Lopes et al. (2006)	10 (7)		7 (5)							
Marec-Berard et al. (2005)	2 (7)				5 (18)		6 (22)			
Oğuz et al. (2004)	1 (3)	0 (0)					20 (56)	16 (44)	24 (67)	9 (25)
Näsman et al. (1994)	20 (26)						15 (20)	0 (0)

Microdontia was recorded in all studies, in a prevalence that ranged from as low as 6% to as high as 65% for CCS. Half of these studies had a healthy control and the prevalence ranged from 0 to 14%. Enamel developmental defects were also recorded in five studies with the prevalence for CCS being around 23% in all but one study (Oğuz et al. 2004) where the prevalence was 56%. This wide range was also seen in the healthy controls for which the prevalence ranged from 0 to 44%.

#### Late defects recorded radiographically

**Fig. 5:**
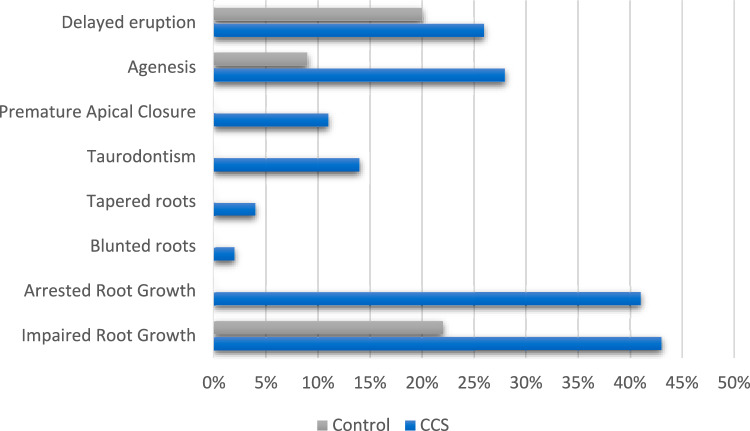
Overall prevalence of dental late defects recorded radiographically in all included studies

Radiographic defects were recorded in 11 studies (Table 9), in a prevalence that ranged from 11% for premature apical closure to > 40% for impaired or arrested root growth for CCS and from 9% for agenesis to 22% for impaired root growth for healthy controls (Fig. 5). Impaired root growth and agenesis were the two defects mostly recorded in included studies. Impaired root growth in CCS was reported in percentages up to 28% in half of the studies while the percentage in the rest of the studies ranged from just below 50% (44%) up to 84%. Corresponding prevalence for healthy controls was much lower and ranged from 19 to 39%. The range for the prevalence of agenesis was not as wide for both CCS and healthy controls, although the latter showed lower percentages.

**Table 9:** Frequency (number of patients) and prevalence of radiographic dental late effects as reported in included studies

Authors/year	Impaired root growth		Arrested root growth		Blunted roots		Tapered roots		Taurodontism		Premature apical closure		Agenesis		Delayed eruption	
	No. of patients (%)		No. of patients (%)		No. of patients (%)		No. of patients (%)		No. of patients (%)		No. of patients (%)		No. of patients (%)		No. of patients (%)	
	CCS	Control	CCS	Control	CCS	Control	CCS	Control	CCS	Control	CCS	Control	CCS	Control	CCS	Control
Proc et al. (2021)	15 (25)															
Shum et al. (2020)	19 (28)												9 (13)			
Kilinc et al. (2019)	24 (26)	–														
Proc et al. (2016)	7 (12)	15 (3)											19 (31)	48 (9)		
Bagattoni et al. (2014)	21 (84)	10 (39)											4 (16)	2 (8)		
Nemeth et al. (2013)	20 (53)	–											18 (47)	2 (5)	6 (16)	–
Cubukcu et al. (2012)	26 (86)	7 (19)											6 (16)	0 (0)		
Lopes et al. (2006)					2 (2)		5 (4)		19 (14)				8 (6)			
Marec-Berard et al. (2005)			12 (44)													
Oğuz et al. (2004)	16 (44)	7 (19)									2 (6)	0 (0)	16 (44)	7 (19)	7 (19)	9 (25)
Näsman et al. (1994)			28 (37)								15 (20)		22 (30)

#### Dental maturity

Dental maturity was recorded in four studies where researchers recorded the significance of deviations between dental and chronological age both within and between study and control group. Results are contradictory as two studies reported an overestimation of dental age by 2 to 4 months in one study (Mitsea et al. 2022) and almost 1 year in the other (Proc et al. 2021) and the other two report an underestimation of 2 months to 1 year (Flores et al. 2015; Bagattoni et al. 2014). Respectively the same researchers found an overestimation of 3–6 months and an underestimation of 5 months for the control groups. The above differences were considered significant except for the study by Mitsea et al. (2022) where results did not show any significant difference in chronological-dental age in neither paediatric cancer group of health controls.

#### Salivary glands

Two studies reported on the effect of antineoplastic treatment on salivary gland functions. Nemeth et al. (2014), reported a 0.85 ml/min stimulated flow rate for CCS and 1.13 ml/min for heathy controls, with the corresponding values for unstimulated flow rate being 0.28 ml/min and 0.38 ml/min, respectively. In the same study 18% of CCS had medium buffer capacity and 82% high, while 57% of the control group had medium and 40% high buffer capacity. In the study by Näsman et al. (1994), flow rate was 1.3 ml/min for healthy controls and 1.1 ml/min for those patients that have undergone only chemotherapy and 0.7 ml/min for those that have undergone HSCT. Thirty-six percent of the survivors that have been treated with chemotherapy and 42% of those that have undergone HSCT had a pH < 4.5, as compared to only 4% of the healthy controls. Regarding microbial counts, CCS had increased counts of *S. mutans* (42% for CHX and 30% for HSCT) and *Lactobacilli* (26% for CHX and 32% for HSCT) compared to only a small 4% of healthy controls that had microbial counts > 10^6^/ml saliva.

#### Risk factors

Six of the included studies recorded possible correlations between defects and specific disease and treatment characteristics. Regarding diagnosis Kilinc et al. (2019) and Näsman et al. (1994) reported no effect on dental caries or crown defects. Proc et al. (2016) reported a positive correlation between age at diagnosis and presence of microdontia, which is in accordance to Bagattoni et al. (2014) that reported an increased risk for microdontia, agenesis and root defects in patients treated at ages < 3 years. Regarding the effect of different treatment protocols Kılınç et al. (2019) and Cubukcu et al. (2012) reported no effect, although the first reported that root malformations are more common in the patients that undergo concomitant radiation therapy and with doses > 20 Gy. Cubukçu and Sevinir (2008) earlier reported a positive correlation between radiation therapy and mean dmft values regardless of the dose and duration. Näsman et al. (1994) finally reported that patients that have undergone HSCT presented dental disturbances more frequently except for hypoplasia.

#### Secondary outcomes

Regarding secondary outcomes, they were recorded only in one study (Shum et al. 2020), where participants with agenesis had a higher mean value (7.9) on the Oral Health Impact Profile compared to those without such a defect (3.7) with the difference being statistically significant (p < 0.05). Also, patients that presented with microdontia were more likely to report “Fairly Often” and “Very Often” in more than 1 domains of the profile almost three times as frequently as those who did not. Although no statistically significant differences were calculated for counts of “Fairly Often” and “Very Often” reporting with any of the dental late effects.

### Quantitative synthesis

A meta-analysis of the included studies’ effect estimates was not regarded as appropriate in the context of the present review. Case–control studies are generally considered to be vulnerable to a higher risk of bias than cohort studies; cases and controls may not have equal opportunities for the ascertainment of exposure, rendering this type of studies more susceptible to selection and recall bias. The same applies for cross-sectional studies. This may, in turn, introduce different biases in meta-analyses of case–control, cross-sectional and cohort studies. Observational studies, irrespectively of how large or well-designed and conducted are, may be subject to biases, in particular, selection in case–control and retrospective cohort studies and observation in case–control as well as retrospective and prospective cohort studies. An overall summary estimate obtained by this review would probably overestimate the real association between exposure and outcome due to the presence of confounding. The level of adjustment for known and putative risk factors that are associated with the outcome varied across the studies and hence, it was not possible to fully take into account their possible impact on the association. The comparison of the crude and multiple-adjusted estimates of the association from the same study population was not possible among the included studies.

### Rehabilitation

Long-term follow-up of dental management was evaluated in six case reports and involved dental treatment of nine CCS with evident dental late effects. Table 10 presents the main characteristics and the treatment followed for each case report according to the late effects detected. Survivors age at presentation varied from 6 to 20 years, age at diagnosis between 2 months and 8 years with rhabdomyosarcoma being the most common diagnosis. Treatment involved chemotherapy in all but two case reports in which only surgical excision was performed (Liu et al. 2021; Kotsiomiti et al. 2013). Five case reports had undergone concomitant radiotherapy (King 2019; Michalak et al. 2019; Zwetchkenbaum and Oh 2007) and two case reports HSCT (Zwetchkenbaum and Oh 2007; King 2019). Regarding the dental effects reported were caries, root abnormalities and agenesis, while orthodontic problems, underdevelopment of the maxilla and the residual ridge and periodontal problems were also detected in a few case reports.

**Table 10:** Patient characteristics, dental late effects and related dental treatment undertaken in included case studies

Author/year	Age at presentation	Diagnosis	Age at diagnosis	Treatment	Dental late effects	Dental rehabilitation	Comments
Chang and Lin (2021)	11 years male	Stage III embryonal parameningeal RMS with intracranial extension	7.5 years	CHX RDT	Multiple caries Crown opacity, hypoplastic teeth Foreshortened and blunted roots, V-shaped roots, impaired root growth, premature apical closure	Extraction, banding, composite resin restorations, root canal fillings, and stainless steel crowns An interim denture to preserve existing dentition for masticatory efficiency and improve aesthetics	6 years post-treatment he developed bony defects Orthodontic treatment and orthognathic surgery not recommended due to susceptibility to pathological mobility and further root resorption morphology 32 years after the initial treatment, multiple teeth loss and severe facial deformity
Liu et al. (2021)	8 years male	Multicystic ameloblastoma of the right mandible	8 years	Surgery and immediate reconstruction using a single left lateral rib graft	Anodontia in the site of surgery	7 months postoperatively, a distraction device placed Once bony consolidation was completed, the distraction device was removed and implants were placed simultaneously Fixed dental bridge was placed	Full osseointegration achieved without complications
King (2019)	18 years female	Stage IV NBL in the thorax with liver metastases	10 months	CHX HSCT	Mild crowding Class III malocclusion Agenesis Delayed eruption All erupted teeth had normal crown morphology, colour and enamel consistency Atypical root morphology Narrow roots in maxillary canines and incisors	Orthodontic treatment contraindicated Direct composite veneers	
King (2019)	15 years male	Head and neck RMS	3 years	CHX RDT Surgical excision	All erupted teeth had normal crown morphology, colour and enamel consistency Upper laterals increased mobility, bleeding on probing, a 5 mm periodontal pocket Atypical root morphology with narrow, tapered roots	Extraction of 22, immediate replacement using a cantilever fibre-reinforced composite (FRC) resin retained bridge (RRB) At 1-year follow-up, to prevent overloading of the already compromised maxillary dentition, a gingival veneer was constructed with 22 and 23 pontics incorporated into the design	Although the patient was satisfied with the aesthetics, the gingival veneer did not fulfil his functional requirements during mastication. Therefore, a maxillary partial cobalt–chrome denture overlaying and clasping all posterior teeth was constructed to replace 22 and 23
King (2019)	20 years female	RMS of the right ocular muscle	10 months	CHX RDT Surgical excision	Underdeveloped maxilla Normal development of mandible Caries Asymptomatic periapical periodontitis #35	A milled implant-retained bar with two locator attachments	No improvement in retention and further endosseous implants placed
Michalak et al. (2019)	8.3 years male	RMS in the nasal part of the throat, the paranasal sinuses, and partially in the orbits and the middle cranial fossa	2 years	CHX RDT Surgical excision	Asymmetry of the middle and lower face Hypoplasia of the maxilla and the mandible Agenesis Arrested root growth Central incisors with grade III mobility Reduction of mandibular abduction to 25 mm	Orthodontic block appliance to increase the range of mandible Abduction	6 months post-treatment, the range of mandible abduction had improved to 33 mm
Michalak et al. (2019)	6 years male	Stage IV Burkitt’s lymphoma	4 years	CHX	Atrophy of the alveolar process of the maxilla and the alveolar part of the mandible Shortened lower face Agenesis Narrow, V-shaped roots Premature apical closure	Prosthesis to restore biting and chewing functions, improve aesthetics and enable better pronunciation	6 months post-treatment, the lower appliance showed poor stability due to the growth of the patient and the eruption of molars At the maturity of 7, eruption of teeth 46, 36 and 26 had begun, with minor signs of disturbed enamel mineralisation in the form of small white spots
Kotsiomiti et al. (2013)	7 years female	Melanotic neuroectodermal tumour of infancy	2 months	Surgery	Severe hypodontia Underdeveloped residual ridge	Series of interim removable partial dentures until end of the growth period 13 years the patient started orthodontic to correct malocclusion and align maxillary teeth	Prosthodontic intervention aimed at improving comfort and aesthetics and preventing further deterioration of the oral structures and function
Zwetchkenbaum and Oh (2007)	12 years male	Stage IV NBL	2 years	CHX RDT TBI + HSCT	Extensive caries Anterior teeth mobility Gingival inflammation Impaired root growth Agenesis	Extractions Maxillary complete denture Implant-retained mandibular overdenture	12 yrs later the patient reports good function and no discomfort Only complaint poor retention

*HSCT* haemopoietic stem cell transplantation, *RMS* rhabdomyosarcoma, *NBL* neuroblastoma, *CHX* chemotherapy, *RDT* radiotherapy, *TBI* total body irradiation

Restorative treatment in combination with prosthodontic rehabilitation was chosen in most case reports, to improve function, preserve bony structures and improve aesthetics. Dental implants and implant-retained dentures were also used in many case reports even for younger survivors. It should be noted that prosthesis reported for the case reports of young survivors were in the form of interim solutions as they were occasionally replaced to accommodate growth and engage erupted mandibular permanent teeth for retention.

Regarding orthodontic treatment conclusion is not clear as in two case reports (Chang and Lin 2021; King 2019) authors suggested that such treatment was contraindicated while in another case report the patient underwent orthodontic treatment to correct malocclusion (Kotsiomiti et al. 2013). Although it should be noted that in the case reports where treatment was contraindicated, patient presented with severe root defects while in the second case report hypodontia and underdeveloped residual ridge were only detected. Chang and Lin (2021) reported that 32 years after end of treatment the patient who initially presented with severe root defects had lost multiple teeth and suffered severe facial deformity, further supporting his initial position regarding orthodontic treatment.

Long-term follow-up of the patients indicated the progressive effects of the disease and its treatment, making rehabilitation challenging.

## Discussion

Early diagnosis and contemporary advances regarding cancer treatment modalities have increased the 5-year survival rate of childhood cancer survivors. This is related to an increasing percentage of children that present with at least one late effect in any organ because of the disease and its treatment (Oeffinger et al. 2006; Blaauwbroek et al. 2007). The effects on dental tissues and the craniofacial complex are detrimental and can cause anatomic, functional, and aesthetic sequelae, as they affect occlusion and facial development. Therefore, early diagnosis, detection of the defects and their long-term monitoring is essential for effective treatment planning to reduce the side-effects of cancer treatment. In addition, counselling of the patient and their caretakers is also very important to improve their quality of life.

The aim of this systematic review was to summarise the existing knowledge on prevalence of oral, dental, and craniofacial side-effects of antineoplastic treatment in CCS in the context of paediatric dentistry. Seventeen retrospective cross-sectional and case–control studies published from 1994 until 2022, were included from the retrieved studies, yielding a total of 983 CCS that were examined clinically and radiographically for any dental adverse effects and compared with 1266 healthy age and gender matched controls. The main finding of this review was that the prevalence of both clinical as well as radiographical dental late defects were very high among childhood cancer survivors compared to healthy controls. Overall, one third of CCS experienced at least one late effect, with corresponding value for the control group being below 25% in most cases. Root abnormalities and agenesis were the two most common defects recorded among all patients examined.

Specifically, regarding oral health three studies reported that CCS are more likely to develop dental caries (Wogelius et al. 2008; Proc et al. 2019; Patni et al. 2023), as mean dmft/DMFT value for CCS was higher when compared to the healthy controls. This is in accordance with the findings from previous studies recording worse clinical indices for CCS (Pajari et al. 1995; Singh et al. 1996; Avşar et al. 2007; Proc et al. 2019). Higher dmft/DMFT scores in CCS may be the effect of reduced salivary secretion and of the microbial shift towards a more cariogenic microflora (Seremidi et al. 2023; Gawade et al. 2014). Furthermore, precious studies also showed that younger patients who receive high doses of radiation are at increased risk of developing tooth decay (Jaffe et al. 1984; Pajari et al. 1995; Kaste et al. 1997; Seremidi et al. 2023). In this review, one study demonstrated a positive correlation between radiation therapy and dental caries (Cubukçu and Sevinir 2008).

Similarly, oral hygiene and gingival indices were worse in CCS, findings that is in accordance to a recent review, presenting increased plaque accumulation and gingivitis for these patients as compared to controls (Busenhart et al. 2018). Researchers associated it with specific phases of the antineoplastic treatment, where patients with low thrombocyte levels are refrained from toothbrushing to avoid bacteremia (Lockhart et al. 2008). Although this discontinuation of toothbrushing is not shared by other researchers, who support that patients should be able to perform oral hygiene procedures without bleeding at widely different levels of platelet counts (da Fonseca 2004).

Prevalence of oral health indices can be affected by confounding factors that cannot be controlled (e.g. frequency and efficiency of brushing, sugar consumption, saliva quality and quantity, etc.) and therefore, the association with specific treatment characteristics is not clear. It is certain that the alterations caused by the antineoplastic medicaments administered during treatment can affect the incidence, but the direct relationship and the degree of the effect cannot be justified, underlying the necessity for proper specialised oral counselling during all stages of treatment.

Late clinical dental developmental defects were documented in 11 studies. In CCS, discolouration was the most common defect followed by microdontia, hypodontia, and enamel disturbances. The overall prevalence of late clinical defects in controls was low. Enamel developmental defects, detected clinically as enamel opacities, are caused by alterations in ameloblast reproduction during tooth formation expressed by secretory function, membrane permeability, and calcium exchange across the cell membrane (Goho 1993). Because of the short half-life of most chemotherapeutic agents used, defects are caused by changes in the function of odontoblasts rather due to their death and are therefore more localised (Avşar et al. 2007).

Defects recorded radiographically were reported in 11 studies and showed that arrested root development was the most prevalent defect followed by agenesis and delayed eruption. The corresponding prevalence in healthy controls was much lower. The range of prevalence of agenesis was less wide in both CCS and healthy controls. However, the percentage of the latter was low. Previous studies have shown that dental development defects, including microdontia, oligodontia, hypodontia, enamel defects, and root malformations, can occur in CCS (Kilinc et al. 2019; Tanem et al. 2022; Halperson et al. 2022; Seremidi et al. 2023). The prevalence of these defects may depend on the type of cancer and the treatment received. Radiation therapy can significantly impair tooth development (Blaauwbroek et al. 2007; Collett and Thonard 1965). The effects of chemotherapy on tooth development still need to be elucidated due to its multi-drug nature and possible differences in the cytotoxic effects of individual chemotherapeutic agents (Jodlowska et al. 2022).

Incidence and severity of these defects depend on risk factors associated with the specific features of antineoplastic therapy. Such a risk factor is age at diagnosis, which is directly related to the stage of tooth development, type, and duration of treatment, absorbed dose and radiation field (Scully and Epstein 1996; Cheng et al. 2000; Seremidi et al. 2019).

Six of the included studies evaluated potential correlations between defects and specific disease and treatment characteristics. For diagnosis Kilinc et al. (2019) and Näsman et al. (1994) reported no effects on crown defects. Proc et al. (2016) reported a positive correlation between age at diagnosis and the presence of microdontia, consistent with Bagattoni et al. (2014) who reported an increased risk of microdontia, aplasia, and root defects in patients treated with doses > 20 Gy.

Cancer therapy can have an impact on dental maturity although how dental maturity is influenced by cancer therapy remains unclear and this reflects the results of the included studies in this systematic review. Dental maturity was assessed in four studies, and investigators documented the significance of deviations in dental age and chronological age within study groups and between study and control groups. The results from the included studies are contradicting since two studies overestimate dental age and two underestimate (Mitsea et al. 2022; Proc et al. 2021; Flores et al. 2015; Bagattoni et al. 2014). The same researchers both overestimate and underestimate dental age for the control group, respectively. Newer evidence supports that there is only small correlation between dental maturity and physical development, with the former only slightly related to skeletal maturation and craniofacial growth (Kanbur et al. 2006).

Saliva production and secretion are important for maintaining a good oral health and function. Therefore, complications resulting from salivary dysfunction such as caries, increased difficulty in swallowing, chewing and speech, can lead to an impaired quality of life. In this systematic review two studies reported the effect of anti-tumour treatments on salivary gland function. Nemeth et al. (2014) reported a lower saliva flow rate (stimulated and unstimulated) in CCS compared to controls. In the same study, 18% of CCS had moderate buffering capacity and 82% had high buffering capacity, whereas 57% of controls had moderate buffering capacity and 40% had high buffering capacity. In a study by Näsman et al. (1994), the unstimulated saliva rate was lower in patients that had stem cell transplantation and received radiation therapy compared to healthy controls and patients only receiving chemotherapy. No difference was seen regarding salivary pH between chemotherapy-treated patients and those who underwent stem cell transplantation and radiation therapy.

Regarding, secondary outcome only one study (Shum et al. 2020) reported oral health-related quality of life. Participants with agenesis had a significant higher mean value on the Oral Health Impact Profile compared to those without agenesis indicating a worse OHRQoL. Also, patients that presented with microdontia were more likely to report “Fairly Often” and “Very Often” in more than 1 domain of the profile, although no statistically significant differences were calculated for counts of “Fairly Often” and “Very Often” reporting with any of the dental late effects. In another study (Wogelius et al. 2011), results show that children with cancer rate their OHRQoL better or equal to those without cancer and that cancer and cancer treatment during childhood is not associated with a decreased OHRQoL. Stolze et al. (2020), reviewed the impact of haematological malignancies on OHRQoL in both adults and children. No robust conclusions could be made regarding the global OHIP-14 score but among OHIP-14 domains, functional limitations and physical pain were given the highest score while social handicap and social disability were given the lowest (Stolze et al. 2020).

Finally, head and neck cancer can lead to physical, physiological, and social problems such as craniofacial deformities in patients (Pertschuk and Whitaker 1985). To solve these problems, depending on the patient's condition, the dentist may consider orthodontic and prosthodontic treatment with surgical intervention. Long-term follow-up of oral rehabilitation was evaluated in six case reports, including nine CCS with severe dental sequelae after cancer treatment (Liu et al. 2021; Kotsiomiti et al. 2013; King 2019; Michalak et al. 2019; Zwetchkenbaum and Oh 2007; Chang and Lin 2021). The dental sequelae reported in the nine case reports were dental caries, root abnormalities, aplasia and underdevelopment of the maxilla and the alveolar ridge. In some case reports, periodontal disease has also been noted. In most case reports, restorative treatments combined with prosthetic rehabilitation were chosen to improve function, preserve bone structure, and improve aesthetics. Dental implants and implant-supported dentures were also used in many case reports of young survivors. Note that prostheses reported in young survivor case reports were a form of interim solution, as they were sometimes replaced to accommodate growth.

Concerning orthodontic treatment, the conclusions are ambiguous, as in two case reports (Chang and Lin 2021; King 2019) the authors suggested that such treatment was contraindicated. In a third case report, the malocclusion was treated with orthodontics (Kotsiomiti et al. 2013). However, when treatment was contraindicated, the patient showed severe root defects or agenesis in combination with an underdeveloped alveolar ridge. Long-term follow-up of patients revealed progressive effects of disease and its treatment, making oral rehabilitation difficult.

### Strengths and limitations

The review tried to present an evidence-based overview of the defects associated to cancer and its treatment in the craniofacial complex with its major strength being the strict inclusion criteria imposed and its broad spectrum of defects assessed in a relatively homogenous manner. Adding evidence to the three previous systematic reviews it attempted to report importance of paediatric dentists in the oncology team, especially during the active cancer treatment to manage acute complications but also when late-stage complications occur.

However, results should be interpreted with caution before any specific conclusion can be drawn due to limitations of the included studies. Language and study design, with a non-randomised sample increases risk of selection and reporting bias. Case–control studies are generally considered to have a higher risk of bias than cohort studies since cases and controls may not have equal opportunity to determine exposure, making these types of studies susceptible to selection and recall biases. The same applies to cross-sectional studies. This can lead to various biases in meta-analyses of case–control, cross-sectional and cohort studies.

Limited comparisons between the included studies could be made due to the heterogeneity of the samples included both regarding disease diagnosis (type and stage of cancer) and treatment characteristics (treatment protocols, duration of treatment, stem cell transplantation). Also, included studies were observational, presenting the subjective perception of each researcher due to the lack of specific indices to categorise and quantify the defects further increasing the risk of overreporting. Finally, pre-existing defects and confounding factors, factors that play a crucial role in the outcome, were not controlled in the included studies.

### Future research

Advanced research should focus on correct screening and early identification of survivors at risk for developing dental late defects. Further evidence is needed to investigate dental late effects, both regarding prevalence and severity, as well as associated risk factors among survivors.

The beneficial effect of individualised pre-screening and preventive dental care must be investigated. Pre-treatment evaluation, evaluation at the end of antineoplastic treatment and long-term monitoring of survivors will allow for more clear conclusions on the effects of treatment on dental structures. Early screening and education of parents and health care providers should aim at improving survivors’ perceived quality of life.

Future studies should also focus on the relationship between specific aspects of HRQoL and disease and treatment-related factors for overall well-being to be achieved. Given that the effects produced by the disease and its treatment vary in extent and severity, it is important to identify the domains that are mainly affected and to achieve satisfaction in those that are important to everyone. Finally, investigation of the empirical relation between physical and psychological variables of HRQoL and cancer survivorship, could contribute to the development of effective psychosocial interventions.

The long-term progression of these defects should also be evaluated. Furthermore, the effect of different oral care and dental treatment protocols on the defects to offer evidence regarding long-term stability through specific guidelines for the long-term follow-up of these patients should be evaluated.

Finally, dentists’ and other healthcare providers knowledge on survivor’s dental care should be assessed, underlining the importance of the multidisciplinary approach and the early and precise involvement of the dentist in the oncological team.

## Conclusion

CCS carries the risk of developing dental sequelae due to the disease and its treatment. The type of defect seems to be related to stage of odontogenesis without the factors affecting their severity not being defined. Most common defects detected were microdontia, impaired root growth and agenesis, with the effect of treatment not being estimated.

It is imperative that regular routine evaluations are performed to assess the development of CCS and overall oral health during the patient’s life span. Also, early diagnosis of late effects, will allow for precise and early consultation and individualised treatment planning.

## Appendix 1

**Table:**

Search number	Query	Filters	Results
1	cancer[Title/Abstract] OR oncolog*[Title/Abstract] OR antineoplast*[Title/Abstract] OR malignan*[Title/Abstract] OR neoplasm*[Title/Abstract] OR tumor[Title/Abstract] OR carcinom*[Title/Abstract]		3,636,091
2	(cancer[Title/Abstract] OR oncolog*[Title/Abstract] OR antineoplast*[Title/Abstract] OR malignan*[Title/Abstract] OR neoplasm*[Title/Abstract] OR tumor[Title/Abstract] OR carcinom*[Title/Abstract]) AND (dent*[Title/Abstract] OR tooth[Title/Abstract] OR teeth[Title/Abstract] OR oral[Title/Abstract])		123,833
3	(child*[Title/Abstract] OR adolescen*[Title/Abstract]) AND ((cancer[Title/Abstract] OR oncolog*[Title/Abstract] OR antineoplast*[Title/Abstract] OR malignan*[Title/Abstract] OR neoplasm*[Title/Abstract] OR tumor[Title/Abstract] OR carcinom*[Title/Abstract]) AND (dent*[Title/Abstract] OR tooth[Title/Abstract] OR teeth[Title/Abstract] OR oral[Title/Abstract]))		4342
4	(child*[Title/Abstract] OR adolescen*[Title/Abstract]) AND ((cancer[Title/Abstract] OR oncolog*[Title/Abstract] OR antineoplast*[Title/Abstract] OR malignan*[Title/Abstract] OR neoplasm*[Title/Abstract] OR tumor[Title/Abstract] OR carcinom*[Title/Abstract]) AND (dent*[Title/Abstract] OR tooth[Title/Abstract] OR teeth[Title/Abstract])		1153
5	(child*[Title/Abstract] OR adolescen*[Title/Abstract]) AND ((cancer[Title/Abstract] OR oncolog*[Title/Abstract] OR antineoplast*[Title/Abstract] OR malignan*[Title/Abstract] OR neoplasm*[Title/Abstract] OR tumor[Title/Abstract] OR carcinom*[Title/Abstract]) AND (dent*[Title/Abstract] OR tooth[Title/Abstract] OR teeth[Title/Abstract])	Humans	904
6	(child*[Title/Abstract] OR adolescen*[Title/Abstract]) AND ((cancer[Title/Abstract] OR oncolog*[Title/Abstract] OR antineoplast*[Title/Abstract] OR malignan*[Title/Abstract] OR neoplasm*[Title/Abstract] OR tumor[Title/Abstract] OR carcinom*[Title/Abstract]) AND (dent*[Title/Abstract] OR tooth[Title/Abstract] OR teeth[Title/Abstract]) AND ((humans[Filter]) AND (english[Filter]) AND (allchild[Filter] OR adolescent[Filter]))) NOT (review[Title/Abstract] OR editorial[Title/Abstract] OR mice[Title/Abstract] OR animal[Title/Abstract])		592
7	(child*[Title/Abstract] OR adolescen*[Title/Abstract]) AND ((cancer[Title/Abstract] OR oncolog*[Title/Abstract] OR antineoplast*[Title/Abstract] OR malignan*[Title/Abstract] OR neoplasm*[Title/Abstract] OR tumor[Title/Abstract] OR carcinom*[Title/Abstract]) AND (dent*[Title/Abstract] OR tooth[Title/Abstract] OR teeth[Title/Abstract])	Humans Child: birth–18 years Adolescent: 13–18 years English	700
8	(child*[Title/Abstract] OR adolescen*[Title/Abstract]) AND ((cancer[Title/Abstract] OR oncolog*[Title/Abstract] OR antineoplast*[Title/Abstract] OR malignan*[Title/Abstract] OR neoplasm*[Title/Abstract] OR tumor[Title/Abstract] OR carcinom*[Title/Abstract]) AND (dent*[Title/Abstract] OR tooth[Title/Abstract] OR teeth[Title/Abstract])	Humans Adolescent: 13–18 years Child: birth–18 years	779
9	(child*[Title/Abstract] OR adolescen*[Title/Abstract]) AND ((cancer[Title/Abstract] OR oncolog*[Title/Abstract] OR antineoplast*[Title/Abstract] OR malignan*[Title/Abstract] OR neoplasm*[Title/Abstract] OR tumor[Title/Abstract] OR carcinom*[Title/Abstract]) AND (dent*[Title/Abstract] OR tooth[Title/Abstract] OR teeth[Title/Abstract])	Humans Child: birth–18 years	779
10	(child*[Title/Abstract] OR adolescen*[Title/Abstract]) AND ((cancer[Title/Abstract] OR oncolog*[Title/Abstract] OR antineoplast*[Title/Abstract] OR malignan*[Title/Abstract] OR neoplasm*[Title/Abstract] OR tumor[Title/Abstract] OR carcinom*[Title/Abstract]) AND (dent*[Title/Abstract] OR tooth[Title/Abstract] OR teeth[Title/Abstract])) AND (surviv*)		179
11	(clinical oncology[MeSH Terms]) AND (dent*[Title/Abstract] OR tooth[Title/Abstract] OR teeth[Title/Abstract])		73
